# Characterization of V1/V2-specific antibodies present in broadly neutralizing plasma isolated from HIV-1 infected individuals

**DOI:** 10.1186/1742-4690-9-S2-P92

**Published:** 2012-09-13

**Authors:** C Krachmarov, K Revesz, R Prattipati, C Reichman, B Li, C Derdeyn, J Sarlo, B Zingman, W Honnen, A Pinter

**Affiliations:** 1 PHRI/UMDNJ, Newark, NJ, USA; 2 Emory Vaccine Center, Atlanta, GA, USA; 3 Montefiore Medical Center, Bronx, NY, USA

## Background

Recent studies of antibodies in human plasma from HIV-1-infected and immunized individuals have revealed an important role for the V1/V2 region of gp120 in an antiviral response, and recent evidence that protection in the RV144 vaccine trial correlated with the presence of V1/V2-specific antibodies suggests that the V1V2 region is an important target for candidate HIV-1 vaccines. However, the function of V1V2-specific antibodies is not well understood. In this study we present data about the development, subtype specificity, and neutralization activity of such antibodies present in plasma from several hundred infected North American and African subjects.

## Methods

Human plasma from infected individuals were screened for neutralization activity versus a panel of subtype B, subtype C, and other Tier 2 pseudoviruses, and were titrated for binding activity against the consensus subtype B, C and A/E V1/V2 fusion glycoproteins, expressed via fusion to a fragment of the MuLV gp70 sequence. This system expresses V1/V2 domains in their native glycosylated and conformational forms. V1V2-specific antibodies were isolated from selected plasma by immunoaffinity chromatography on gp70-V1/V2 antigen columns, and characterized for neutralizing activity against various HIV-1 pseudoviruses.

## Results

Most (>80%) of the HIV-1 infected subjects possessed robust levels of V1/V2 binding activity versus the three antigens. Interestingly, the development of V1/V2-reactive antibodies tracked with the development of autologous neutralizing antibodies in several subjects infected with subtype C viruses. Immunoaffinity-purified V1/V2-specific antibodies from selected broadly neutralizing plasma samples also possessed broad neutralization activities, with IC_50_s generally in the 1-20 μg/ml range.

## Conclusion

Highly cross-reactive V1V2-specific antibodies were present in almost all broadly neutralizing human plasmas at large concentrations, and frequently possessed modest neutralizing activities against a range of isolates, including tier 2 viruses. Additional information about the nature of these antibodies and their target epitopes would help elucidate their potential roles in protection against infection.

